# Supplementary material for: Comparison of chemical compounds associated with sclerites from healthy and diseased sea fan corals (Gorgonia ventalina)
Source: PeerJ. 2017 Aug 25;5:e3677. doi: 10.7717/peerj.3677 (PMC5572935; doi:10.7717/peerj.3677)
Supplement: Table S1 [file peerj-05-3677-s001.docx]

**Table S1:** List of semivolatile organic compounds (SVOCs) isolated from sclerites from: healthy fans (HH), from healthy tissue from diseased fans (HD) and diseased tissue (DD) and their respective roles.

| **SVOCs** | **Sclerites** | | | **Biological** | **Reference** |
| --- | --- | --- | --- | --- | --- |
|  | **HH** | **HD** | **DD** | **Function** |  |
| Bis( 2-ethylhexyl) phtalate | ✓ |  |  | Apoptosis inductor | Priya & Jayachandran, 2012 |
| 2,3-dihydroxypropyl ester Octadecanoic acid | ✓ |  |  | Unknown |  |
| 3,6-dione- (5,17,20) Cholestane- | ✓ |  |  | cytotoxic | Ktari, Blond, & Guyot, 2000 |
| Dodecyl acrylate | ✓ |  |  | anti-oxidant | Joo , Kim & Lee, 2010 |
| 2-Hexadecanol | ✓ |  |  | Anti-microbial, anti-oxidant | Senthil et al., 2016 |
| 2-methyl-2-Undecanethiol | ✓ |  |  | Anti-microbial | Meenakshi et al., 2013 |
| 5-Octadene (E) | ✓ |  |  | Unknown |  |
| 2-Tetradecene | ✓ |  |  | Anti-microbial | Roy et al., 2010 |
| 1-Tridecene | ✓ |  |  | Unknown |  |
| 5,22-dien-3-ol, (3·)-Cholesta |  | ✓ |  | Unknown |  |
| 2-ethyl-1-Decanol |  | ✓ |  | Anti-microbial | Kumar et al., 2013 |
| 15-Heptadecenal |  | ✓ |  | Unknown |  |
| 7-Hexadecene |  | ✓ |  | Anti-microbial | Mujeeb, Bajpai & Pathak, 2014 |
| 2,6,10,14-Hexadecatetraen-1-ol, 3,7,11,15-tetramethyl-acetate |  | ✓ |  | Unknown |  |
| Methyl stearate |  | ✓ |  | Unknown |  |
| 2-methyl-1-Dodecanol |  | ✓ |  | Anti-microbial | Kuppuswamy, Jonnalagadda, & Arockiasamy, 2013 |
| 9-methyl-1-Undecene |  | ✓ |  | Antibacterial | Okwu & Ighodaro, 2010 |
| 4-Nonene 2-ethyl |  | ✓ |  | Unknown |  |
| Phytol acetate |  | ✓ |  | Unknown |  |
| Tetradecane |  | ✓ |  | Unknown |  |
| 3-Tetradecene |  | ✓ |  | Unknown |  |
| 4-Tetradecene |  | ✓ |  | Unknown |  |
| 5-Tetradecene |  | ✓ |  | Unknown |  |
| 3,7,11,15-Tetramethyl-2-hexadecen-1-ol |  | ✓ |  | Unknown |  |
| 2-Tridecanol |  | ✓ |  | Unknown |  |
| 5-Tridecene |  | ✓ |  | Unknown |  |
| 1-Undecanol |  | ✓ |  | Unknown |  |
| Bis (7-methyloctyl) ester Phthalic acid |  |  | ✓ | Anti-oxidant | Sivasubranian & Brindha, 2013 |
| 5-Cholestene-3-ol, 24-methyl |  |  | ✓ | Unknown |  |
| 1-Decene |  |  | ✓ | Unknown |  |
| 4-Decene |  |  | ✓ | Unknown |  |
| 5-Eicosene |  |  | ✓ | Unknown |  |
| 4-Ethyl-2-octanol |  |  | ✓ | Unknown |  |
| Heptadecane |  |  | ✓ | Unknown |  |
| Hexadecanoic acid, 2-hydroxy-1-(hydroxymethyl)ethyl ester |  |  | ✓ | anti-oxidant | Kumar et al., 2013 |
| 2-Hexyl-1-octanol |  |  | ✓ | anti-oxidant | Shah & Ahmed, 2016 |
| 2-methyl Nonadecane |  |  | ✓ | Unknown |  |
| 2-Methyl-1-undecanol |  |  | ✓ | Unknown |  |
| 7-methyl-undecene |  |  | ✓ | Unknown |  |
| 2-Propyl-1-pentanol |  |  | ✓ | Anti-microbial-anti-fungal | Pattnaik et al., 1996 |
| 2-Tetradecanol |  |  | ✓ | Unknown |  |

**Table S1 (continue)**

| **VOCs** | **Sclerites** | | | **Biological** | **Reference** |
| --- | --- | --- | --- | --- | --- |
|  | **HH** | **HD** | **DD** | **Function** |  |
| 2-Butyl-1-decene | ✓ | ✓ | ✓ | Unknown |  |
| Campesterol | ✓ | ✓ | ✓ | Antioxidant | Senthil, Rameashkannan, & Mani, 2016 |
| Cetene | ✓ | ✓ | ✓ | Unknown |  |
| Cholesta-4,6-dien-3-ol, (3) | ✓ | ✓ | ✓ | Product of the oxidation of Cholesterol | Liu &Shan, 2006 |
| Cyclopropane, 1-(2-methylbutyl)-1-(1-methylpropyl) | ✓ | ✓ | ✓ | Anti-fungal | Sheoran et al., 2014 |
| 1-Decanol, 2-hexyl | ✓ | ✓ | ✓ | Anti-microbial | Xiangwei et al., 2006 |
| Dodecane, 2,6,11-trimethyl | ✓ | ✓ | ✓ | Unknown |  |
| 1-Dodecene | ✓ | ✓ | ✓ | Anti-microbial | Senthil, Rameashkannan & Mani, 2016 |
| 17-(1,5-Dimethylhexyl)-10,13-dimethyl-2,3,4,7,8,9,10,11,12,13,14,15,16,17-tetradecahydro-1H-cyclopenta[a]phenanthren-3-ol | ✓ | ✓ | ✓ | Anti-microbial | Altameme Hameed & Kereem, 2015 |
| Ergosta-5,22-dien-3-ol, (3·,22E,24S | ✓ | ✓ | ✓ | Apoptsis | Byju et al., 2014 |
| Nonadecane | ✓ | ✓ | ✓ | Anti-fungal | Omoruyi, Afolayan & Bradley,  2014 |
| 3-Octadecene, (E) | ✓ | ✓ | ✓ | Unknown |  |
| 1-Tetradecene | ✓ | ✓ | ✓ | Unknown |  |
| Benzoic acid, 4-ethoxy-, ethyl ester | ✓ | ✓ |  | Anti-microbial | Sheela & Uthayakumari, 2013 |
| Cholesterol | ✓ | ✓ |  | Cell membrane |  |
| Ergost-5-en-3-ol, (3) | ✓ | ✓ |  | Anti-oxidant | Ponnamma & Manjunath, 2012 |
| Hexadecanoic acid, methyl ester | ✓ | ✓ |  | Anti-fungal |  |
| 1-Octanol, 2-butyl | ✓ | ✓ |  | Unknown |  |
| 4-Trifluoroacetoxytridecane | ✓ | ✓ |  | Unknown |  |
| Cholest-5-en-3-ol, 24-propylidene-, (3·) |  | ✓ | ✓ |  |  |
| Glycerol 1-palmitate |  | ✓ | ✓ | Unknown |  |
| Undecane, 2-methyl |  | ✓ | ✓ | Unknown |  |
| Cholestane-3,6-dione, (5) | ✓ |  | ✓ |  |  |
| 3-Hexadecene, (Z) | ✓ |  | ✓ | Unknown |  |
| 1-Undecene, 7-methy | ✓ |  | ✓ | Unknown |  |

**References list for supplementary table**

Altameme H, Hameed I, Kereem M. 2015. Analysis of alkaloid phytochemical compounds in the ethanolic extract of *Datura stramonium* and evaluation of antimicrobial activity. *African Journal Biotechnology* 14: 1668-1674.

Byju K, Anuradha V, Vasundhara G, Nair SM Kumar NC. 2014. In vitro and in silico studies on the anticancer and apoptosis-inducing activities of the sterols identified from the soft coral, *Subergogia reticulata*. *Pharmacognosy Magazine* 10.4103/0973-1296.127345.

Joo SS, Kim YB, Lee DI. 2010. Antimicrobial and antioxidant properties of secondary metabolites from white rose flower. *The Plant Pathology Journal* 26:57-62.

Ktari L, Blond A, Guyot M. 2000. 16β-Hydroxy-5α-cholestane-3,6-dione, a novel cytotoxic oxysterol from the red alga *Jania rubens. Bioorganic & Medical Chemistry Letters* 10: 2563-2565.

Kumar S, Samydurai P, Ramakrishman R, Nagarajan N. 2014. Gas chormatography and mass spectrometry analysis of bioactive constituents of *Adiantum capillus-veneris* L. *International Journal of Pharmacy and Pharmaceutical Sciences* 6:60-63.

Kuppuswany KM, Jonnalagadda B, Arockiasamy S. 2013. GC-MS analysis of chloroform extract of *Croton bonplandianum*. *International Journal of Pharma and Bio Sciences* 4: 613-617.

Liu ZQ, Shan HY. 2006. Cholesterol, not polyunsaturated fatty acid, is target molecule in oxidation induced by reactive oxygen species in membrane of human erythrocytes. *Cell Biochemistry and Biophysics* 45:185-193.

Meenakshi VK, Gomathy S, Senthamarai S, Paripooranaselvi, Chamundeswari KP. 2012. GC-MS determination of the bioactive componenst of *Microcosmus exasperatus* Heller, 1887. *Journal of Current Chemical and Pharmaceutical Science* 2:271-276.

Mujeeb F, Bajpai P, Pathak N. 2014. Phytochemical evaluation, antimicrobial activity and determination of bioctive components form leaves of *Aegle marmelos*. *BioMed Research International* 10.1155/2014/497606.

Priya MA, Jayachandran S. 2012. Indcution of apoptosis and cell cycle arrest by Bis (2-ethylhexyl) phthalate produced by marine *Bacillus pumilus* MB 40. *Chemico-Biological Interactions* [10.1016/j.cbi.2011.11.005](http://dx.doi.org/10.1016/j.cbi.2011.11.005).

Okwu DE, Ighodaro BU. 2010. GC-MS evaluation of bioactive compounds and antibacterial activity of the oil fraction from the leaves of *Alstonia boonei* De Wild. *Der Pharma Chemica* 2: 261-272.

Omoruyi BE, Afolayan AJ, Bradley G. 2014. Chemical composition profiling and antifungal activity of the essential oil and plant extracts of *Mesembryanthemum edule* (L.) *bolus* leaves. *African Journal of Traditional, Complementary and Alternative Medicine* 11:19-30.

Ponnamma SU, Manjunath K. 2012. GC-MS analysis of phytocomponents in the methanolic extract of *Justicia wynaadensis* (Nees) T Anders. *International Journal of Phama and Bio Sciences* 3:P570-P576.

Priya AM, Jayachandran S. 2012. Induction of apoptosis and cell cycle arest by Bis (2-ethylhexyl) phthalate produced by marine *Bacillus pumilus* MB40. *Chemico-Biological Interactions* 10.1016/j.cbi.2011.11.005.

Sarada K, Margret RJ, Mohan VR. 2011. GC-MS determination of bioactive components of *Naringi crenulata* (Roxb) Nicolson. *International Journal of ChemTech Research* 3:1548-1555.

Senthil JS, Rameashkannan MV, Mani P. 2016. Phytochemical profiling of ethanolic leaves extract of *Ipomoea sepiraria* (Koenig Ex. Roxb). *International Journal of Innovative Research in Science, Engineering and Technology* 10.15680/IJIRSET.2016.0503063.

Sethi A, Praskash R, Shukla AD, Bhatia A, Singh RP. 2013. Identification of phytochemical constituents from biologically active pet ether and chloroform extracts of the flowers of *Allamanda violacea* A.DC (Apocynaceae). *Asian Journal of Pant Science and Research* 3:95-108.

Shah WA, Ahmed A. 2016. Scientific validation of *Salix caprea* inflorescence and chemical composition, pharmacological potential of its aromatic water. Journal of Pharmaceutics and Drug Delivery Research. 10.4172/2325-9604.C1.012.

Sheela D and F Uthayakumari. 2013. GC-MS analysis of bioactive constituents from coastal sand dune taxon – *Sesuvium portulacastrum* (L.) *Bioscience Discovery* 4:47-53.

Sheoran N, Nadakkakath V., Munjal V, Kundu A, Subaharan K., Venugopal V, Rajamma S, Eapen S, Kumar A. 2015. Genetic analysis of plant endophytic *Pseudomonas putida* BP25 and chemo-profiling of its antimicrobial volatile organic compounds. *Microbiological Research* 173: 66-78.

Sivasubranian R, Brindha P. 2013. In vitro cytotoxic, antioxidant and GC-MS studies on *Centratherum punctatum* Cass. *International Journal of Pharmacy and Pharmaceutical Sciences* 5:364-367.

Roy S, Rao K, Bhuvaneswari Ch, Giri A, Mangamoori LN. 2010. Phytochemical analysis of *Andrographis paniculata* extract and tis antimicrobial activity. *World Journal of Microbiology and Biotechnology* 10.1007/s11274-009-0146-8.

Xiangwei Z, Xiaodong W, Peng N, Yang P, Jiakuan C. 2006). Chemical composition and antimicrobial activity of the essential oil of *Sagittaria trifolia*. *Chemistry of Natural Compounds* 42: 520-522.
